# Anomalous Influence of Salt Concentration on Deposition of Poly(l-Lysine)/Cellulose Sulfate Multilayers Evidenced by In Situ ATR-FTIR

**DOI:** 10.3390/molecules25102336

**Published:** 2020-05-16

**Authors:** Martin Müller

**Affiliations:** 1 Department Functional Colloid Materials, Leibniz Institute of Polymer Research Dresden, Hohe Straße 6, 01069 Dresden, Germany; mamuller@ipfdd.de; 2 Department Chemistry and Food Chemistry, Technical University of Dresden, 01062 Dresden, Germany

**Keywords:** polyelectrolyte multilayers, poly(l-lysine), cellulose sulfate, deposition, ATR-FTIR

## Abstract

The deposition of polyelectrolyte (PEL) multilayers (PEMs) of poly(l-lysine)/cellulose sulfate (PLL/CS) onto germanium (Ge) substrates depending on salt concentration (c_S_) and deposition step z at constant PEL concentration c_PEL_ = 0.01 M and pH = 7.0 was studied. In situ ATR-FTIR spectroscopy was used for the quantitative determination of alternate PLL/CS deposition profiles (adsorbed amount versus z) and total deposited PEM amount. By varying c_S_ from 0 M to 1.0 M, a maximum of deposited amount was obtained at 0.1 M, so that both no salinity (0 M) and high salinity (1.0 M) revealed deposited amounts that were far lower than for mean salinity (0.1 M). Furthermore, in situ ATR-FTIR allowed to determine the detailed modulation of the PEL composition during the consecutive PEM deposition, which was interpreted as being due to both diffusion of given PEL from the PEM interior towards the outermost region and release of the PEM upon contact with the bulk oppositely charged PEL solution. Finally, ex situ ATR-FTIR measurements on the PEL solutions after deposition of PEM-20 revealed the distinct release of PEL from the PEM solely for c_S_ = 1.0 M, due to the highest mobility of PEL under high salt conditions. These studies help to prepare functional PEM coatings with defined thicknesses and morphologies for the passivation and activation of material surfaces in the biomedical and food field.

## 1. Introduction

The fabrication of polyelectrolyte (PEL) multilayers (PEMs) is based on the consecutive adsorption of polycations (PC) and polyanions (PA), typically on charged substrates beginning with the oppositely charged PEL, but also possibly on neutral substrates. PEM were introduced by Decher [1] and have been present in research in the colloid and surface science field, as well as in numerous applications in the life sciences and in the biomedical field. Concerning applications, the PEM or LbL concept has been used for the robust modification of planar, curved and porous substrates based on aqueous systems related to biomedicine [2,3], sensorics [4,5] and separation technology [6,7]. Studies on PEM still present challenging topics like growth mechanisms, the location of the counterions, PEL composition and nano-/micro- structures in the bulk and surface phases, all of which are influenced by external parameters (salt, pH, polymer concentration, temperature) and PEL structures, as documented by past and recent reviews [8,9,10,11,12].

**Figure 1:**
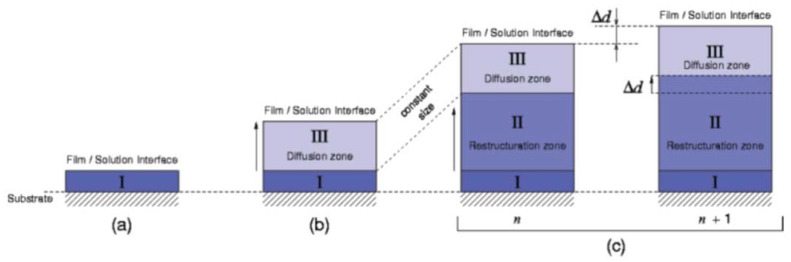
Schematic representation of a polyelectrolyte (PEL) multilayer (PEM), fabricated by consecutive deposition of polycations and polyanions taken from Reference [17]. (Reproduced with kind permission of ACS). Meanings of zones I, II and III are explained in the text below. (**a**), (**b**) and (**c**) denote early, medium term and late period of PEM growth, *n* the PEL deposition step and Δ*d* denotes the thickness increment per *n*.

Herein, we report on a fundamental study on PEM growth mechanism based on two analytically accessible PEL. Recently, in the same context, we reported on the experimentally observed huge thickness increase with increasing adsorption steps for the poly(ethyleneimine)/poly(acrylic acid) (PEI/PAA) system, and pointed out that various models for PEM growth describing the relationship between adsorbed amount and adsorption step z prevail [13]. In one of the very first experimental works on the PEM of poly(allylamine) and poly(styrene sulfonate) (PAH/PSS), a linear relationship was observed, suggesting a well-defined regular PEL uptake and increase with additive thickness increments [1]. Later, exponential relationships were experimentally obtained for the first time [14] when e.g., charged polypeptides like poly(l-lysine) (PLL) and poly(l-glutamic acid) or polysaccharides like hyaluronic acid (HYA) were used, and the growth model was refined so that the differential additive thickness increments were no longer constant, but became dependent on adsorption step z. A three-zone model of PEMs including loosely structured zones for inner surface (I) and outer surface zone (III), and a more tightly structured core zone II between zones I and II, was first postulated by Ladam [15], and later refined by Porcel [16,17], the latter of which is given in the following scheme of Figure 1 and briefly described in the following.

In the early period (a) deposition of the first layers (deposition steps *n* = 1, 2, 3 …) is highly dependent on the substrate surface properties forming an initial zone I. From a certain n onward, on top of zone I a loose zone III is formed, in which PELs are assumed to be rather diffusive, so that PELs supplied from the solution phase are uptaken but may “diffuse in and out” of zone III. During this medium term period (b) PEM deposition shows an exponential dependence on n until a certain thickness is reached. Thereafter, PEM deposition shows a linear dependence on n. During this late period (c) it is assumed, that “diffusion” zone III saturates keeping a constant thickness and that between zones I and III a new growing zone II is formed [16,17,18,19]. According to these authors supplied PELs still “diffuse in” but can no longer “diffuse out” and stay at the bottom of zone III in a complexed state, starting to form the rigid “restructurisation” zone II. After this point, newly supplied PELs are uptaken at the top of zone III, but for every uptaken PEL, another leaves zone III at the bottom and contributes additively to the “restructurisation” zone II, growing from then on in a linear fashion.

Somewhat related to this aspect of the “diffusing in and out” of PELs at PEM, Hoogeveen and Kovacevic [20,21] introduced another aspect. Based on reflectometric studies on the consecutive deposition of the polycation PDMAEMA and polyanion PAA, these authors observed deposition profiles with significant zig/zag-like features. These composition modulations were interpreted by the mutual pull-out of the already adsorbed PELs by the supplied oppositely-charged PELs in all adsorption steps. In principle, this is not in contradiction to the “diffusing in and out” concept mentioned above, but rather, it is the extreme case, where a PEL already integrated in PEM does not only “diffuse out” but is even pulled out and leaves the PEM upon electrostatic interaction with the supplied oppositely charged one.

It is well known, that salt influences electrostatically driven PEL diffusion scenarios in PEMs, since the Debye length l_D_ (electrostatic reach) is significantly lowered. Note that for 0.1 M monovalent salt concentrations, l_D_ is around 1 nm. Earlier studies of such salt screening effects on PEM were given by von Klitzing [22] and Schlenoff [23]. In the inner study, strong polyanions and strong polycations with varying charge densities were used. For high charge densities, a monotonous increase with increasing salinity was obtained by scaling with c_S_^1/2^, which was not observed for low charge densities. In contrast, in the latter report PEM composed of a weak polyacid (PAA) and a strong polycation (PDADMAC) showed a nonmonotonous increase featuring a maximum of deposition at medium c_S_ = 0.3 M, while lower and higher c_S_ resulted in lower deposition. A model based on ion exchange and swellability was used for explanation. The topic is still under debate; a recent study by Tang [24] on the effect of salt on model PEM reported linear growth regimes for low and exponential regimes for high salt concentration due the increased diffusion propensity of PEL at high salinity. Very recently, the ionic strength and temperature effects on the hydration of PEM were reported by Lutkenhaus [25], describing how water molecules are distributed around ion pairs in microenvironments of PEM using in-situ ATR-FTIR spectroscopy.

Herein, the effects of salinity on PEM are revisited, and the influence of sodium chloride concentration (c_NaCl_) on the deposition profile (adsorbed amount versus adsorption step z) and PEL composition is addressed. The PEM system consisting of the polycation poly(l-lysine) and the polyanion cellulose sulfate (PLL/CS) was chosen for two reasons. On the one hand, for analytical reasons, since PLL and CS can be conveniently detected via FTIR spectroscopy based on intense Amide I and Amide II bands (PLL) and intense ν(SO_2_) or ν(C–O) stretching bands (CS). Attenuated total reflexion (ATR) Fourier transform infrared (FTIR) spectroscopy was applied due to its surface sensitivity, in order to characterize this PEM system with respect to adsorbed amount and composition as introduced earlier [26,27]. On the other hand, the PLL/CS system contains biorelated PEL and may therefore be envisaged as a biomedical coating system for studies and applications including protein interaction or drug delivery. Therefore, this study helps to improve biorelated PEM systems as a modification strategy of biomaterials and medical devices in contact with biofluids, since salt concentration is an easily controllable experimental parameter. 

This paper is structured as follows: First, in-situ ATR-FTIR data on PEM deposition according to adsorption step z = 1–20 at three different salt concentrations, i.e., c_NaCl_ = 0, 0.1 and 1.0 M, are given. Second, ex situ-ATR-FTIR data on the composition of PLL and CS adsorbing solutions after PEM-20 deposition are presented. Finally, a growth mechanism of PEMs of PLL/CS under the given conditions considering adsorption and desorption is suggested and discussed.

## 2. Results and Discussion

### 2.1. Deposition of PEM PLL/CS Based upon Salinity

#### 2.1.1. In Situ ATR-FTIR Spectra

**Figure 2:**
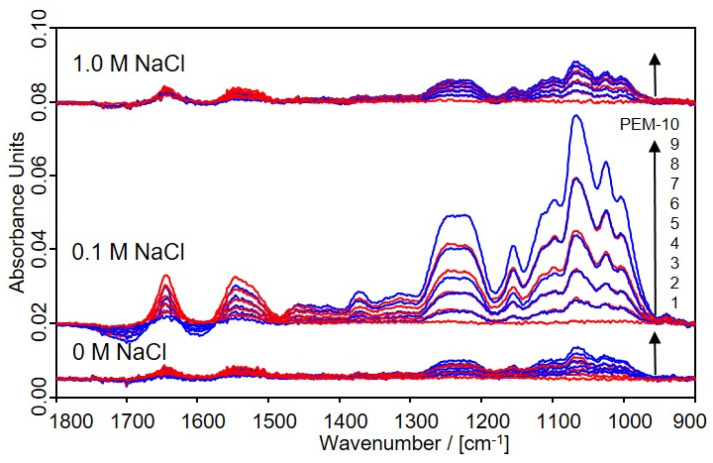
In situ ATR-FTIR spectra of the consecutive deposition of PEM from solutions of PLL and CS at pH = 7.0 for c_PEL_ = 0.01 M and c_NaCl_ = 0, 0.1 and 1.0 M (from bottom to top) onto Ge-IRE. PEM-z are shown from z = 1 to 10 adsorption steps from bottom to top (red: PLL steps, blue: CS steps). Typical IR bands of PLL and CS used for further analysis are described in the Table 1.

In the Figure 2, typical in situ ATR-FTIR spectra of the consecutive adsorption of PLL and CS from 0.01 M solutions at pH = 7.0 and c_NaCl_ = 0, 0.1 and 1 M (left to right) for a single PLL layer (z = 1, PEM-1, bottom, red), a double PLL/CS layer (z = 2, PEM-2, blue) and triple PLL/CS/PLL layers (z = 3, PEM-3, red) of up to 10 consecutively adsorbed PLL and CS layers (z = 10, PEM-10, blue) are shown.

In these PEM spectra, the increasing overall intensity and the changes of both the Amide I and Amide II bands at around 1640 and 1550 cm^−1^ due to PLL and of ν(SO_2_) band at 1248/1222 cm^−1^ (doublet), and of the ν(C–O) band at 1050 cm^−1^ due to CS in relation to z, are most significant. Furthermore, an increasing negative ν(OH) band at around 3400 cm^−1^ with increasing z shows up, which is not given herein but in the Figure S1 of the Supplementary Materials, and has been discussed previously for another PEM system [13]. The diagnostic IR bands are assigned in the following Table 1.

#### 2.1.2. ATR-FTIR Deposition Profiles

**Figure 3:**
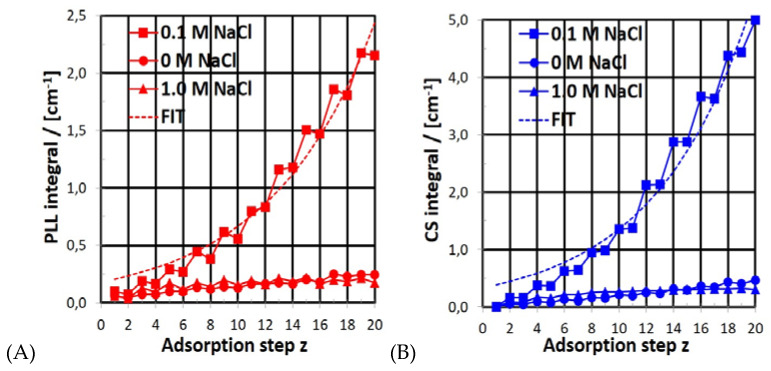
Variation of band integrals A_PLL_ (red, **A**) and A_CS_ (**B**, blue) related to the in situ ATR-FTIR spectra given in Figure 2 in relation to z = 1–20) at c_PEL_ = 0.01 M and pH = 7.0 for c_S_ = 0, 0.1 and 1.0 M.

To gain a more detailed picture, the integrated areas (A) of the IR bands introduced above (Table 1) were used to quantify PEM-PLL/CS deposition. This quantitative analytical approach is justified, since the deposited PEM-20 films do not exceed a thickness of d = 200 nm, which classifies PEM-20 films as thin films. To check this point critically, low resolution SFM images on locally scratched PEM-20 films deposited at c_S_ = 0.1 M were recorded, and are provided in Figure S3 of the Supplementary Materials. In these images, a film thicknesses of d = 44 ± 7 nm was observed. The classified thin films have the analytical advantage that the integrated area of a given band has a linear relation to the adsorbed amount; therefore, plots of the integrated area versus adsorption step z are directly related to plots of adsorbed amount versus z, which is the film growth. Since PLL and CS have different IR bands, their contributions can be separated and their compositions determined for every adsorption step z. In detail, the deposited PLL amount was quantified using areas of the diagnostic Amide II band at around 1550 cm^−1^, further denoted as A_PLL_, while the deposited CS amount was quantified using areas of the ν(SO_2_) band at 1225 cm^−1^, denoted as A_CS_. The ATR-FTIR-based PLL and CS deposition profiles for PEL concentration c_PEL_ = 0.01 M (PLL, CS) and salt concentrations c_NaCl_ = 0, 0.1 and 1.0 M are given in the Figure 3.

For both A_PLL_ (A) and A_CS_ (B), significant nonlinear increases of the deposited amount in relation to z were obtained if a medium salt concentration c_NaCl_ = 0.1 M was applied. However, for both A_PLL_ and A_CS_, significantly small linear increases of the deposited amount in relation to z were found, if either no (0 M) or a high (1.0 M) salt concentration was applied. Plots of the IR band integral A versus adsorption step z were fitted by an exponential growth function for PLL (A_PLL_) and CS (A_CS_), respectively:

A(z) = A_0_ exp(a z) (1)

Convenient fits were obtained for PLL (Figure 3A, broken red line) using parameters A_0_ = 0.529 cm^−1^ and a = 0.366 cm^−1^ and for CS (Figure 3B, broken blue line) using parameters A_0_ = 0.529 and a = 0.366 cm^−1^. All of the observed growth parameters, i.e., A_0_ and a, for PLL and CS obtained for c_NaCl_ = 0, 0.1 and 1.0 M are summarized in the Table 2.

The exponential growth found by us for the PLL/CS system has also been reported for similar PEM systems like PLL/hyaluronic acid (HYA) and PLL/poly(l-glutamic acid) (PLG) [14,15,16,17,18,19]. The authors of these reports claimed that the internal diffusion of polyelectrolytes within the porous PEM volume phase was the main factor of exponential growth in PEM systems, as described in the Introduction. In detail, it was claimed that polyanions migrate in the direction of the last adsorbed polycation layer at the solid PEM/liquid polycation interface, while polycations migrate in the direction of the last adsorbed polyanion layer at the solid PEM/liquid polyanion interface. This means that with increasing PEM thickness (increasing z), the diffusion space increases, and thus, an increasingly mobile, oppositely charged polyelectrolyte portion is given the chance to migrate. Consequently, the thickness increment becomes dependent on adsorption step z, and nonlinear growth is obtained.

#### 2.1.3. Modulation of Uptake and Release

ATR-FTIR spectroscopy is sensitive for diffusion processes in PEM films attached to substrates, which are transparent to IR radiation like germanium or silicon crystals. This enabled us to look more closely at the deposition profiles for the three different c_S_. Indeed, slight but significant modulations of A_PLL_ and A_CS_ could be identified. For A_PLL_, the values were always higher at odd steps (z = 1, 3, 5 …) in comparison to even steps (z = 2, 4, 6 …), while for A_CS_, the opposite values were always higher at even steps (z = 2, 4, 6 …) in comparison to odd steps (z = 3, 5, 7 …). This means that whenever PLL is present as the adsorbing solution (odd steps), there is a partial decrease of the previously adsorbed CS portion, while whenever CS is present (even steps), a partial decrease of the previously adsorbed PLL portion is observed.

**Figure 4:**
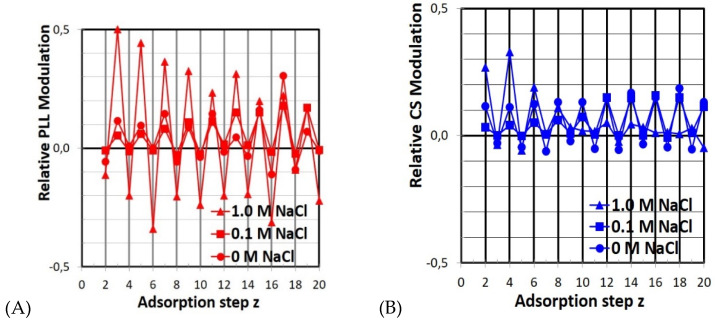
Increases and decreases of relative PLL (**A**) and CS portions (**B**) in relation to adsorption step z with respect to preceding z–1 for z = 2 to 20 (PEM-2 to PEM-20).

In an attempt to quantify empirically the modulation amplitude for the salinities c_NaCl_ = 0, 0.1 and 1.0 M, we calculated the relative changes of the PLL (A_PLL_) or CS portion (A_CS_) in the PEM from one step z to the following step z + 1 for every z. In Figure 4, these relative changes, denoted as relative PLL or CS modulation, are plotted versus adsorption step z.

Significant modulations of PLL or CS portions could be identified for all salinities (0–1.0 M). For PLL, larger modulations were observed compared to CS, meaning that the decrease and increase of PLL portions were more pronounced than for CS. For both PLL and CS, the portion increase was greater than the portion decrease, meaning that there was a net increase of PEL material in any adsorption step z; otherwise, PEM growth would not occur. However, modulations were strongly dependent on salinity. Most significantly, PLL modulations were largest for c_S_ = 1.0 M, but lower for 0.1 M and 0 M NaCl. To obtain an empirical yet quantitative determination of the effect of salinity on the modulation amplitudes, the mean relative modulation amplitude $M_{AVERAGE,\;PLL,\;CS}$ was calculated by summing the magnitudes of all the modulations and then dividing by 19, as follows:

(2) $$M_{AVERAGE,PLL,CS}=\overset{20}{\underset{z=2}{\sum }}\frac{|A_{PLL,CS}\left(z\right)-A_{PLL,\;\;CS}\left(z-1\right){|}_{}}{19}$$

The values of $M_{AVERAGE,\;PLL,\;CS}$ are summarized in Table 2. Obviously, for the PLL portion, the highest modulation of uptake and release (M_AVERAGE_ = 0.257) was found at highest c_S_ = 1.0 M, while for c_S_ = 0 (0.065) and 0.1 M (0.084), lower and similar modulation values were obtained. For the CS portion, the highest modulation was obtained at c_S_ = 0 M (0.094), while lower and similar values were found for 0.1 M (0.054) and 1.0 M (0.070). In both cases, the lowest PLL and CS portion modulation values were found for c_S_ = 0.1 M.

Hence, one can speculate that the observed high PEM deposited amount at a medium salt concentration, i.e., c_S_ = 0.1 M, might be related to the observed low PEM modulation amplitude. According to the literature, salt has a significant influence on both single and consecutive PEL deposition [22,23,28,29,30]. Generally, low and high salinities cause low and high deposited PEL amounts, respectively, for single PEL adsorption. This is due to both the high self-repulsion at the already overcharged surface of like-charged and rather extended PEL conformation (trains) in the case of low salinity, and low self-repulsion at the like-charged surface and rather coiled PEL conformation (loops) in the case of high salinity. However, for consecutive PEL adsorption in relation to salinity, the situation is different and more complex. As pointed out in the introduction, PEL diffusion plays a decisive role in PEM deposition; the PEL present at the outermost PEM layer may not only bind at the outermost oppositely charged PEL layer, but may cause the PEL located in deeper zones of the PEM to migrate to the outermost surface, or may migrate by themselves into deeper zones to compensate for the excess charge. Both PEL structure and salt can have a significant influence on this diffusion scenario, so that, e.g., smaller and hydrophilic PEL may be more diffusive, and the presence of salt reduces the Debye length so much that PEL in PEM are less fixed at their positions and are also diffusive.

Now, to explain our findings of low (frustrated) overall deposition for low and high salinities and high deposition for medium salinity, we assumed mainly electrostatic forces governing intramolecular, intermolecular and interfacial PEL properties within the PEM in terms of PEL conformation, attraction between outermost PEM region and oppositely charged PEL and mobility within the PEM diffusion zone defined in the Introduction. These properties contribute to the observed deposition trends for low, medium and high salinity in the following way. For low salinity (0 M) PEL at PEM are rather stretched forming flat adsorbed layers, are rather strongly bound and show low mobility (“electrostatically fixed”) resulting in low overall PEM deposition. For high salinity (1 M, l_D_ = 0.3 nm) PEL at PEM are rather coiled forming loopy adsorbed layers, are rather weakly bound and show high overall mobility within the PEM resulting also in low adsorbed amount. Whereas for medium salinity (0.1 M, l_D_ = 1 nm) PEL at PEM show moderate stretching, binding and mobility resulting in moderate yet higher PEM deposition compared to low and high salinity. Obviously, it is the balance of all these contributions which favors medium salinity. However presumably mobility is the key property, which should be not too high that PEL might leave the PEM and also not too low, that PEL are not uptaken in the diffusion zone. Furthermore, the different modulation behavior of PLL (0.257) and CS (0.070) for the high salt case (1.0 M) might be a consequence of molecular weight, which for PLL (50,000 g/mol) was smaller than for CS (100,000 gmol). Since it has been suggested that smaller polyelectrolytes are more diffusive than larger ones, we propose that a high salt regime, where PEL are more flexible, selectively determines the molecular sizes. This is not the case for low salt regimes, where PEL are less flexible and in which oppositely charged PEL are rather fixed electrostatically, regardless of molecular sizes.

**Figure 5:**
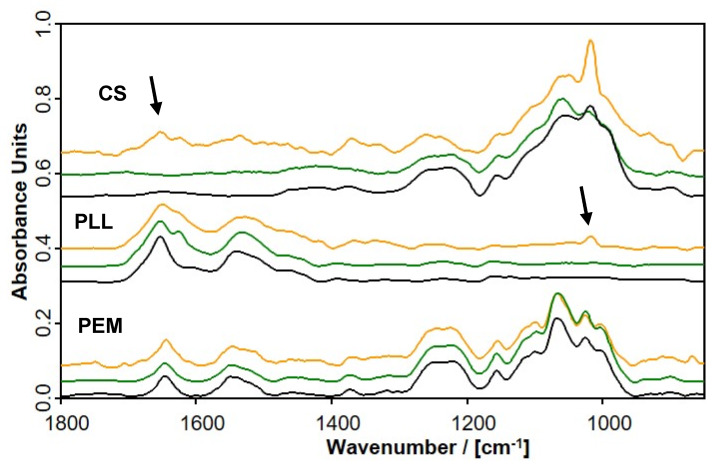
In situ ATR-FTIR spectra of PEM-20 films of PLL/CS (bottom) and ex situ ATR-FTIR spectra of dry films of the used PLL (middle) and CS solutions (top) at pH = 7.0 for c_PEL_ = 0.01 M and c_NaCl_ = 0 M (black), 0.1 M (green) and 1.0 M (orange) after consecutive adsorption at Ge IRE. For these PLL and CS solutions, 50 microliters of PLL and CS solutions, respectively, were spread onto a Ge IRE substrate. Note that the spectra of PEM-20 were scaled by a factor of 10 (0 M) and 15 (1.0 M), while the spectra of the PLL and CS solutions were scaled by a factor of 4 and 10 for comparison purposes. Arrows indicate diagnostic bands of the respective released guest component in the host PEL solution.

Such uptake/release tendencies for PLL and CS are, on the one hand, in line with the results of studies by Kovacevic and Cohen-Stuart [21], who reported similar tendencies for a different system. These authors claimed, that sufficiently high amounts of salt are able to plasticize and cause dissolution and erosion of the actual PEM. This was confirmed by Schlenoff [23], who additionally reported the nonmonotonous effect of salt concentration on PEM deposited amount, resulting in a maximum at medium c_S_ and lower amounts at lower and higher c_S_, confirming our results. On the other hand, our results are seemingly in conflict with the three-zone-model which only allows diffusion of PEL to occur within the PEM, but does not allow them to leave the PEM. However, bridging these views, it has to be noted that the local PEL concentration modulations observed in our ATR-FTIR study may stem either from the loss/uptake of the outermost PEL material, or from the depletion/enrichment of the internal PEL material by diffusion to the outermost/interior PEM regions, as was proposed by e.g., Hübsch et al. [18]. The related analytical modalities concerning ATR-FTIR detection of this scenario are presented in the Figure 5. At present, in situ ATR-FTIR spectroscopy can qualitatively describe PEL composition modulations, but cannot quantitatively separate these two crucial contributions (i.e., loss or depletion by diffusion, uptake or enrichment by diffusion) due to the exponentially scaled evanescent field characteristics of the method (see above). Future studies will address this issue quantitatively.

### 2.2. Composition of PLL and CS Adsorbing Solutions

To further address this issue and check the findings from the in situ ATR-FTIR deposition data concerning the loss/uptake or depletion/enrichment in relation to salinity, we took ex situ ATR-FTIR measurements on 0.01 M PLL solutions and CS solutions after z = 20 consecutive adsorption steps for c_NaCl_ = 0, 0.1 and 1.0 M. A similar experiment was reported recently for a PEM system composed of cationic poly(etyhleneimine) (PEI) and anionic poly(acrylic acid) (PAA) [13]. It was expected that in the FTIR spectra of the host PLL solution, traces of guest CS might be detected, while in those of the host CS solution, traces of guest PLL might be present, indicating either the complete loss of the outermost PEL or the depletion of internal PEL material. The ATR-FTIR spectra recorded from the respective PLL and CS solutions with different salinities, together with the in situ ATR-FTIR spectra of the respective PEM-20 films of consecutively adsorbed PLL/CS, are given in Figure 5.

Comparing the six ex situ FTIR spectra on the dried PLL (middle panel) with the dried CS solutions (top panel) at c_S_ = 0, 0.1 and 1.0 M, some significant trends could be identified. At first, the PLL solutions for c_NaCl_ = 0 M and 0.1 M contained no significant amounts of CS, which can be evidenced by the missing composed band at around 1050 cm^−1^ (ν(C–O)) due to the presence of saccharide hydroxyl and ether group diagnostics for CS. Secondly, the CS solutions for c_NaCl_ = 0 M and 0.1 M did not contain significant amounts of PLL, as shown by the absence of both Amide I and Amide II bands between 1700–1500 cm^−1^. Solely PLL solutions for c_NaCl_ = 1.0 M contained considerable amounts of CS at the limit of FTIR detection, which can be evidenced by the presence of the ν(C–O) band diagnostic for CS. Furthermore, only the CS solutions for c_NaCl_ = 1.0 M contained considerable amounts of PLL, which can be evidenced by the presence of Amide I and Amide II bands between 1700–1500 cm^−1^ and is in line with the highest mean PLL modulation amplitude for c_NaCl_ = 1.0 M (Table 2). Finally, comparing the spectrum of the dried pure CS solution with that of the dried PEM film, no significant shifts of the ν(SO_2_) doublet at 1248/1222 cm^−1^ due to the presence of sulfate groups was observed, as shown in Figure S2 (Supplementary Materials). Hence, the presence of a similar charge state for uncomplexed and ionically interacted CS sulfate groups is concluded.

### 2.3. Growth Mechanism

Finally, a growth mechanism of PEMs of PLL/CS under the given conditions considering either uptake/loss or enrichment/depletion is suggested. Obviously, ex situ ATR-FTIR solution data show that only for c_S_ = 1.0 M, there is mutual physical pull-out of PEL material by the respective oppositely charged one. This finding can be confirmed by the modulation amplitudes given in the Table 2 and discussed above, where for c_S_ = 1.0 M, the largest amplitudes, meaning the largest PEL (PLL) portion loss, prevailed. However, one has to take into account that the modulation amplitudes detected herein can also be caused by the diffusion of PEL from the interior zone to the oppositely charged outermost layer zone. Qualitatively, this can be explained by the general ATR-FTIR detection concept, which is schematically given in the Figure 5. Polymer material or composition is detected most sensitively at the inner substrate/polymer interface, but less sensitively with increasing distance d from this interface to the outer polymer/H_2_O interface, since the electrical field of the evanescent wave scales with an exponentially damped function (A(d) = A_0_ (1 − exp(−d/dp)).

Nevertheless, there was a profound loss of PLL and CS only for c_S_ = 1.0 M, as proven by the ex situ experiments on the respective CS and PLL adsorbing solutions which had been in contact with the PEM-1 to PEM-20 films. This finding was also observed for other systems, like the aforementioned PEI/PAA system [13], where besides diffusion, also escape from the PEM prevailed.

## 3. Materials and Methods

### 3.1. Materials

Commercial poly(l-lysine) (PLL, 70,000–30,000 g/mol) was obtained from Sigma-Aldrich (Darmstadt, Germany) and cellulose sulfate (CS, 100,000 g/mol, degree of substitution d_S_ = 0.5) from Euroferm (Erlangen Germany). All polyelectrolyte (PEL) solutions were prepared by dissolving dry powdered samples of PLL and CS in Millipore water (Merck Millipore, Darmstadt, Germany) at concentrations of c_PEL_ = 0.01 M. The pH was maintained at 7.0 by the addition of small amounts of 0.1 M HCl or 0.1 M NaOH solution. Optionally, NaCl was added to PEL solutions to obtain 0.1 M and 1.0 M NaCl concentrations. Trapezoidal germanium (Ge) internal reflection elements (IRE, 50 × 20 × 2 mm^3^) were purchased from Komlas GmbH (Berlin, Germany). Ge IRE were cleaned by low pressure UV plasma (plasma cleaner PDC-32 G, Harrick, Ossining, NY, USA) to remove contaminants and create reproducible surface properties. After the plasma cleaning step, Ge IRE was immersed in Millipore water at pH = 7.0 for at least 1 h.

### 3.2. PEM Deposition

PEM deposition was studied using the stream coating concept [26]. First, 0.01 M PLL solution with either 0, 0.1 or 1.0 M NaCl (deposition solution), either Millipore water with 0, 0.1 M or 1 M NaCl solution (rinse solution), 0.01 M CS solution with either 0, 0.1 or 1.0 M NaCl (deposition solution), and either Millipore water with 0, 0.1 M or 1 M NaCl (rinse solution) was injected in the respective sequences into the S compartment of the in situ ATR cell. Deposition solutions with 3 mL PLL or CS solution remained in the in situ ATR cell for 5 min, after which they were withdrawn, and rinsing solutions of 5 mL 0, 0.1 or 1.0 M NaCl were added for 1 min, after which they were discarded. In this report, z = 20 consecutive adsorption steps were applied, thereby generating films PEM-z denoted as PEM-1, PEM-2, PEM-3 etc. up to PEM-20.

### 3.3. In Situ Attenuated Total Reflection Fourier Transform Infrared (ATR-FTIR) Spectroscopy

An in situ ATR-FTIR apparatus (Optispec, Zürich, Switzerland) installed on a FTIR spectrometer (IFS 55, Bruker Optik GmbH, Leipzig, Germany) was used for the ATR-FTIR measurements on PEM deposition. This apparatus was based on a special mirror setup and an in situ flow cell (M.M., IPF Dresden e.V.) housing the trapezoidal Ge internal reflection element (Ge IRE). ATR-FTIR spectra were recorded applying the single-beam-sample-reference (SBSR) technique [31], probing separately the upper sample (S) and lower reference (R) half of the Ge IRE (50 × 20 × 2 mm^3^ resulting in N = 11 active reflections on the shorter front side) by the IR beam. Ge IRE was clamped within the in situ cell and sealed by two O-rings on the front and two on the back. The intensities of spectra I_S_(ν) recorded at the upper Ge IRE half (contact with PEL solution with or without salt), and those of spectra I_R_(ν) recorded at the lower Ge IRE half (contact with water with or without salt), were divided, and the absorbance spectra A_SBSR_ = −log (I_S_(ν)/I_R_(ν)) were computed. ATR-FTIR spectra were achieved by coadding 50 scans at a spectral resolution of 2 cm^−1^. ATR-FTIR spectroscopy provided quantitative access to concentration c based on a modified Lambert‒Beer law, given in Equation (3):

*A* = *N ε c d_E_*, (3)

including the integrated absorbance of a given IR band A [cm^−1^], the number of active reflections N, the absorption coefficient ε [cm/Mol], concentration c [mol/cm^3^], and the effective thickness d_E_ [cm^−1^] [32]. From *c*, the surface concentration Γ [mol/cm^2^] can be calculated, if thickness *d* is known:

Γ = *c d*. (4)

An introduction to quantitative application of ATR-FTIR spectroscopy to PEM systems can be found in [27]. The used band areas A_PLL_ and A_CS_ were approximately proportional to the surface concentration of PLL and CS, respectively, for the so called thin film case. Thin films in that sense are defined with respect to the critical thicknesses d_CRIT_ (for Ge around 200–300 nm) which is dependent on, e.g., the depth of penetration d_P_ interrelated with refractive indices, the wavenumber position of the given IR band and the incident angle. For *d* < d_CRIT_, the PEL deposited amount scaled linearly to the measured A_PLL_ or A_CS_. In contrast, for *d* > *d*_CRIT_, the deposited amount no longer scaled linearly, but with a damped exponential function of type (1–exp (*d*/*d*_P_)).

### 3.4. Scanning Force Microscopy (SFM)

An Ultramiscroscope (Nanostation II, Bruker Nano GmbH, Berlin, Germany) was used, consisting of an optical microscope and SFM attachment using silicon probe tips from Nanosensors (Darmstadt, Germany) having apex radii of around 10 nm as cantilevers. PEM films on Si IRE were probed in noncontact mode (topography, error phase mode) and used as cantilevers, operated at frequencies of around 160 kHz and free amplitudes of around 100 nm. Notably, the PEM thickness was measured based on topographical images (32 × 32 μm) within the region of scalpel cuts and then evaluating the line profiles at 30 different steps from undamaged film and bare silicon.

## 4. Conclusions

In situ ATR-FTIR spectroscopy was applied to monitor the consecutive deposition of polyelectrolyte (PEL) multilayers (PEMs) of poly(l-lysine)/cellulose sulfate (PLL/CS) onto germanium (Ge) substrates in relation to the adsorption step z for three different salinities, i.e., 0, 0.1 and 1.0 M, at pH = 7.0.

Analytically valuable deposition profiles based on diagnostic bands for PLL (A_PLL_) and CS (A_CS_) in ATR-FTIR spectra, recorded for every adsorption step z, were obtained.

By varying NaCl concentration c_S_ from 0 M to 1.0 M, a maximum of deposited amount was obtained for c_S_ = 0.1 M, so that both no salinity (0 M) and high salinity (1.0 M) revealed deposited amounts that were far lower than for mean salinity (0.1 M).

**Figure 6:**
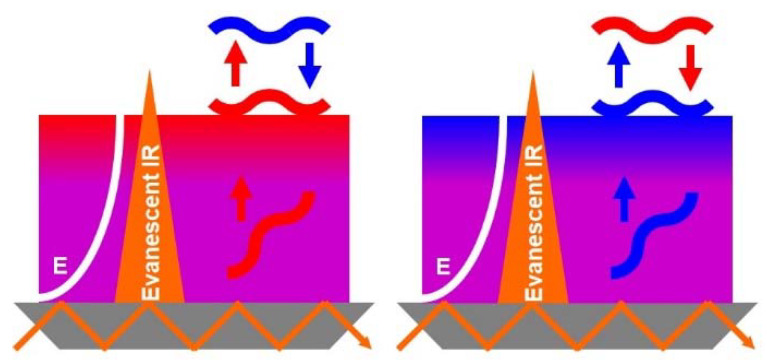
Scheme of the scenario whereby PEM is in contact to either the cationic PLL or anionic CS solution, and of the ATR-FTIR detection modalities concerning diffusion towards or away from the outermost PEM region.

Furthermore, in-situ ATR-FTIR analysis provided evidence for the modulation of local PEL material concentrations (gradient) within PEM for every adsorption step. The detailed scenario is presented in Figure 6, considering the PEM interior (violet) and the outermost PEM region (red or blue) and either the incoming polycations (PLL, red) or polyanions (CS, blue).

In the odd steps (z = 2*n* + 1, for n = 0–9, left side), whenever the PLL solution was present at PEM, the CS moved towards the outermost PEM region, while in the even steps (z = 2*n* + 2, for n = 0–9, right side), whenever CS was present, PLL moved. Again, salinity was shown to have a significant effect on the modulation amplitude of the PLL and CS portion. High salinity (1.0 M NaCl) caused the largest modulation amplitude for PLL and a medium one for CS. In contrast, low (0 M) and medium salinities (0.1 M) caused medium CS modulation amplitudes. 

An ex-situ ATR-FTIR analysis of the adsorbing PLL and CS host solutions after the construction of PEM-20 at the applied salinities revealed no portions of oppositely charged guest PEL species at low (0 M) and medium salinities (0.1 M). In contrast, significant guest CS portions were found in PLL solutions, and significant guest PLL portions were found in CS solutions with high salinity (1.0 M), suggesting not only migration to, but also release away from, the outermost PEM region.

These deposition trends were interpreted considering electrostatic forces affecting intramolecular, intermolecular and interfacial PEL properties, which can be modulated by salt concentration i.e., Debye length. These properties are related to either PEL stretching or coiling by mutual repulsion of like charged segments, higher or lower attraction between outermost PEM region and oppositely charged PEL and lower or higher mobility of PEL within the diffusion zone III (see Introduction) for either lower or higher salt concentration, respectively. Restricting on these three properties the following balances arise for low, medium and high salinity. 

At low salinity, PEL stretching is rather high (“flat adsorbed layers”), attraction between outermost located PEM and PEL is high but mobility within PEM diffusion zone is low due to “electrostatic fixation” resulting in low overall PEM deposition. At high salinity, intramolecular PEL stretching is rather low (“loopy adsorbed layers”), intermolecular attraction low, but mobility within PEM diffusion zone high due to charge screening resulting in low overall PEM deposition. At medium salinity PEL stretching, intermolecular attraction and mobility within PEM are all moderate resulting in moderate overall PEM deposition. Presumably, mobility in the PEM diffusion zone plays a decisive role. It should be not too high, so that PEL can be lost and not too low, so that PEL can not be uptaken within the diffusion zone.

These studies provide information that may be useful in the preparation of polyelectrolyte-based films with controlled thicknesses, nanostructures and rinse stabilities for interactions with biofluids in the biomedical and food fields.

## Figures and Tables

**Table 1:** Assignment of diagnostic IR bands in ATR-FTIR spectra of PLL/CS PEM films given in Figure 1.

Wavenumber (cm^−1^)	Assignment	Component
3700–3100	ν(OH)	H_2_O
1640	Amide I	PLL
1550	Amide II	PLL
1248/1222	ν(SO_2_)	CS
1050	ν(C–O)	CS

**Table 2:** Growth parameters A_0_ and a for individual PLL and CS deposition profiles obtained from fitting the data given in Figure 2 and mean modulation amplitude M_AVERAGE_.

PEL, c_NaCl_	A_0_	a	M_AVERAGE_
PLL, 0 M	0.068 ± 0.005	0.068 ± 0.005	0.084
CS, 0 M	0.062 ± 0.007	0.104 ± 0.007	0.094
PLL, 0.1 M	0.181 ± 0.025	0.130 ± 0.008	0.065
CS, 0.1 M	0.337 ± 0.048	0.139 ± 0.008	0.054
PLL, 1 M	0.107 ± 0.013	0.133 ± 0.018	0.257
CS, 1 M	0.036 ± 0.009	0.051 ± 0.009	0.070

## Supplementary Materials

The following are available online: Figure S1: Overview in-situ ATR-FTIR spectra of PEM films, Figure S2: ATR-FTIR spectra on dried films of PEM and CS, Figures S3 and S4: SFM images on PEM films.
