# Supplementary material for: Anomalous Influence of Salt Concentration on Deposition of Poly(l-Lysine)/Cellulose Sulfate Multilayers Evidenced by In Situ ATR-FTIR
Source: Molecules. 2020 May 16;25(10):2336. doi: 10.3390/molecules25102336 (PMC7288139; doi:10.3390/molecules25102336)
Supplement: Supplementary file 1 [file molecules-25-02336-s001.pdf]

Supplementary Materials

Anomalous Influence of Salt Concentration on Deposition of Poly(L-Lysine)/Cellulose Sulfate Multilayers Evidenced by In-Situ ATR-FTIR

Martin Müller

1. Overview in-situ ATR-FTIR spectra of PEM films

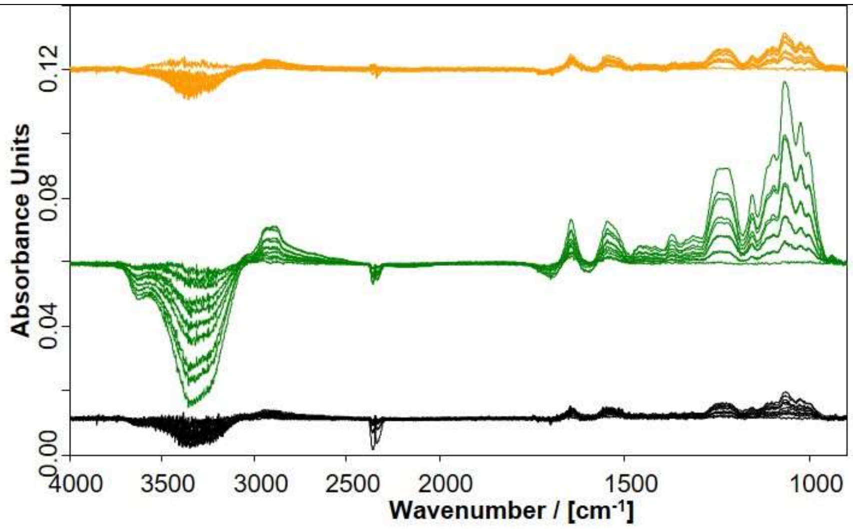

Figure S1. In-situ-ATR-FTIR spectra of PEM-PLL/CS from PEM-1 to PEM-10 for c_{NaCl} = 0 M (black), 0.1 M (green) and 1.0 M (orange). (Negative spikes at around 2200 cm^{-1} are due to incomplete atmospheric carbon dioxide compensation.)

2. ATR-FTIR spectra of dried films of PEM and CS

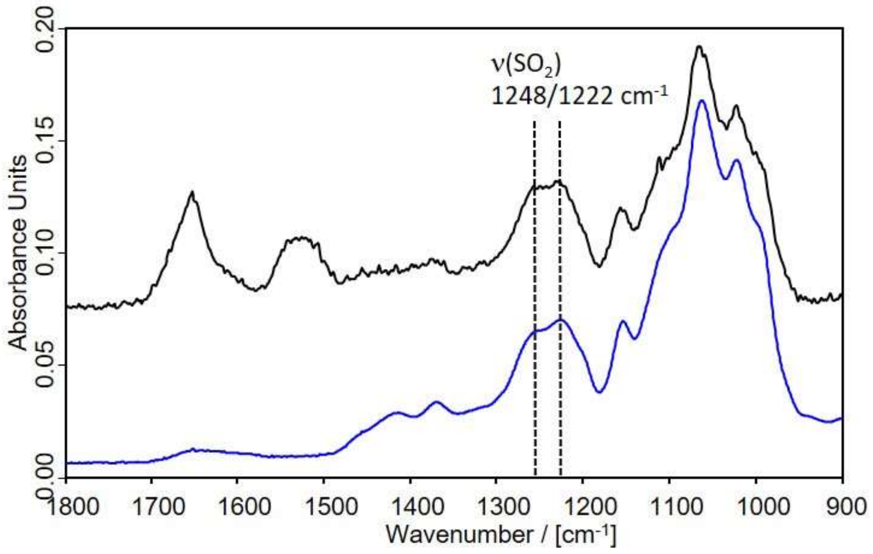

Figure S2. ATR-FTIR spectra of dry films (N_2 purging) of PEM-PLL/CS in comparison to pure CS.

3. SFM images on PEM films

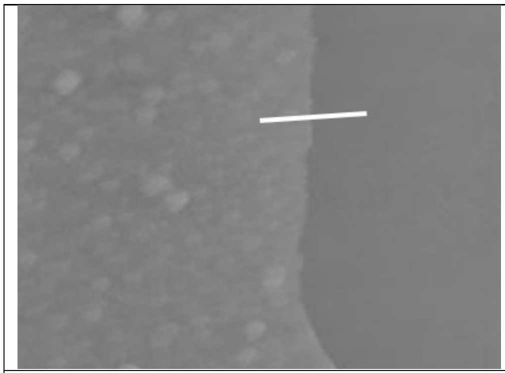

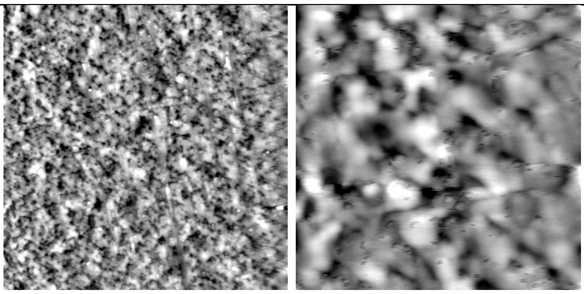

Figure S3. SFM image (16 x 12 μm) of PLL/CS-PEM-20 deposited at c_{NaCl} = 0.1 M at a scratched (medical scalpel) position. The white line indicates a typical position for a profile analysis, from which the step height (PEM film thickness) was determined. Step heights at N = 30 different positions were determined and averaged. A thickness of $d = 44 \pm 7$ nm for PEM-20 at cS = 0.1 M was calculated.

Figure S4. Typical SFM images (left: 10 x 10 μm, right: 2 x 2 μm) of PLL/CS-PEM-20 deposited at c_{NaCl} = 0.1 M.
